# Regulation of ERα Stability and Estrogen Signaling in Breast Cancer by HOIL-1

**DOI:** 10.3389/fonc.2021.664689

**Published:** 2021-05-20

**Authors:** Jianing Ding, Peng Kuang

**Affiliations:** ^1^ Department of Medicine, Queen Mary School, Medical College of Nanchang University, Nanchang, China; ^2^ The Oncology Center, The First Affiliated Hospital of Nanchang University, Medical College of Nanchang University, Nanchang, China

**Keywords:** HOIL-1, ERα, breast tumor, stability, ubiquitin

## Abstract

Estrogen receptor α (ERα) is the major driver for breast tumor carcinogenesis and progression, while ERα positive breast cancer is the major subtype in breast malignancies, which account for 70% breast cancers in patients. The success of endocrine therapy such as tamoxifen is one of the biggest breakthroughs in breast cancer treatments. However, the endocrine therapy resistance is a headache problem in breast cancer. Further mechanisms need to be identified to the effect of ERα signaling in controlling breast cancer progression and drug resistance. HOIL-1 was firstly identified as the ERα transcriptional co-activator in modulating estrogen signaling in breast cancer. In our current study, we showed that HOIL-1, which was elevated in breast cancer, related to good prognosis in ERα positive breast cancer, but correlated with poor outcome in endocrine-treated patients. HOIL-1 was required for ERα positive breast cancer proliferation and clone formation, which effect could be rescued by further ERα overexpression. Further mechanism studies showed that HOIL-1 is required for ERα signaling activity in breast cancer cells. HOIL-1 could interact with ERα in the cytosol and modulate ERα stability *via* inhibiting ERα K48-linked poly-ubiquitination. Thus, our study demonstrated a novel post-translational modification in ERα signaling, which could provide novel strategy for ERα-driven breast cancer therapy.

## Introduction

The ubiquitination process functions to modulate the protein disposal and function in eukaryotic cell hemostasis (1, 2). E3 ubiquitin ligases promote the transfer of ubiquitin from the E2 ubiquitin conjugating enzymes to target protein substrates *via* their lysine residues (3). The ubiquitination can be classifies as several types through the lysine residues on the ubiquitin proteins, including K63, K48, linear ubiquitination and mono-ubiquitination (2). The ubiquitination process was firstly discovered as a target for proteins degradations (4). However, the studies in recent years showed that the ubiquitin systems play important role in protein functions in a group of regulatory pathways, such as signaling transduction, DNA damage response and endocytosis (5, 6).

The ubiquitin process involves the coordinated reactions of E1 ubiquitin-activation enzyme, E2 ubiquitin enzymes and E3 ubiquitin ligases (4). It has been show that the E3 ubiquitin ligases are the key factors, which specifically interact with certain substrates and E2 enzymes for ubiquitin transferring. According to the functional domains of E3 ubiquitin ligases, they can be separated into four groups: HECT (Homologous to the E6-AP Carboxyl Terminus), RING, U-box and PHD-finger family (7). The RING family proteins are composed of more than 700 different proteins, most of which are not well investigated (8). Based on current understanding of RING proteins, the RING family members are involved in several cell physiological functions, including cell proliferation, protein trafficking and DNA repair (9, 10). One of the most thoroughly studied proteins is BRCA1 (RNF53), which participates in DNA repair, gene expression and protein ubiquitination (11–13). In clinics, BRCA1 mutations are proved to be the major driver for familial breast cancer and ovarian cancer (14).

Recent studies showed that RING family proteins play important role in tumor carcinogenesis and progression (15). Several atypical ubiquitination manners, which modified quite a few nuclear receptors, exhibited regulatory functions in cancer signaling transductions. For example, RNF31 and RNF8 function ubiquitin ligases, which promote the monoubiquitination of ERα, enhance ERα protein stability and estrogen signaling activity in breast cancer cancers (16, 17). Our previous studies also showed TRIM56, one of the RING family members, promotes ERα K48-linked ubiquitination and estrogen signaling in breast malignancies (18). In the current study, we identify HOIL-1 (Haem-oxidized IRP2 Ubiquitin Ligase-1) functions as an ubiquitin ligase to modulate ERα protein stability. HOIL-1 is composed of 510 amino acids, which is mainly localized in the cytoplasm in breast cancer cells. HOIL-1 associates with ERα AF1 domain *via* its RING domain and prolongs ERα protein stability, which subsequently enhances ERα target gene expression and breast cancer cell proliferation.

## Materials and Methods

### Cell Culture

MCF-7, T47D and HEK293 cells are originated form American Type Culture Collection (ATCC). T47D cells are cultured with RPMI-1640 (42401, Life Technologies) supplemented with 2 mM L-glutamine (25030, Life Technologies) and 10% FBS. MCF-7 and HEK293 are culture with Dulbecco’s Modified Eagle’s Medium that contains 4.5 g/L glucose and 4 mM L-glutamine (DMEM, 41965, Life Technologies) supplemented with 10% Fetal Bovine Serum (FBS, 10270, Life Technologies). All cell lines are characterized by cell line authentication. The cell line authentication *via* Short Tandem Repeat (STR) is performed *via* PowerPlex 21 system. The STR data of MCF-7 and T47D cell lines are found consistent with STR data in ATCC.

### Plasmids and siRNA

The FLAG-HOIL-1 plasmid is acquired from previous study (19). The HOIL-1 deletion constructs were acquired from the previous study (19). The ERα full and deletion constructs were described in previous study (20). The HA-K48 Ubi, HA-K63 Ubi and HA-Ub-KO plasmids were used in previous study (20). The Estrogen-Response-Element (ERE)-TK reporter and renilla plasmids were used in previous study and are transfected with Lipofectamin 2000 (1662298, Invitrogen) (21). For siRNA transfection, the HOIL-1 siRNA sequences are #1: 5-GCC UUC AGC UAC CAU UGC ATT-3’, 5-UGC AAU GGU AGC UGA AGG CTT-3; #2: 5-CAC ACC UUC UGC AGG GAG UTT-3, 5-ACU CCC UGC AGA AGG UGU GTT-3. The siControl sequences are 5-UUC UCC GAA CGU GUC ACG UTT-3, 5-ACG UGA CAC GUU CG GAGA ATT-3.

### RNA Extraction and qPCR Analysis

RNeasy plus mini kits were used to extract total RNA (Qiagen). Real-time PCR was performed as previously described (18). 36B4 was used as internal control. Primer sequences for qPCR are provided: GREB1 F: CGT GTG GTG ACT GGA GTA GC, R: ACC TCT TCA AAG CGT GTC GT; HOIL-1 F: GCA GAT GAA CTG CAA GGA GTA TCA, R: TGC AGC ATC ACC TTC AGC AT; ER F: GCT ACG AAG TGG GAA TGA TGA AAG, R: TCT GGC GCT TGT GTT TCA AC; 36B4 F: GGC GAC CTG GAA GTC CA ACT, R: CCA TCA GCA CCA CAG CCT TC; PS2 (TFF1) F: TGG GCT TCA TGA GCT CCT TC, R: TTC ATA GTG AGA GAT GGC CGG.

### Quantification of Cell Viability

MCF-7 and T47D cells were transfected with siHOIL-1 or siControl in 24-well plates. Twenty-four hours after transfection, the cells number was countered and 4,000 cells were seeded into 96-well plates. The relative cell viability was measured at indicated time points. Cell numbers were determined using the WST-1 cell proliferation reagent as previously described (22).

### Western Blotting

Cells were harvested and lysed with RIPA buffer. Proteins were separated by electrophoresis on SDS-polyacrylamide gel electrophoresis (PAGE) and electro-transferred to PVDF membrane. The antibodies used in this study were listed here: Anti-ERα (D8H8, 8644, Cell signaling Technology); Anti-ERα (SC-56833, Santa Cruz); Anti-HA (MMS-101R, COVANCE); Anti-myc (9E10, ab32, Abcam); Anti-myc (Ab9106, Abcam); HOIL-1 (Ab108479, Abcam); Anti-Flag (Ab49763, Abcam); Anti-GFP (Ab290, Abcam). Membranes were then washed with PBS for three times and incubated with secondary antibodies Peroxidase-Conjugated AffiniPure Goat Anti-Mouse IgG or Goat Anti-Rabbit IgG. Fluorescent signals were visualized with ECL system (Amersham Imager 600, USA).

### Luciferase Assay

The luciferase activity of estrogen signaling activity was performed using the Dual-Luciferase Reporter kit (Promega, Germany). The ERE luciferase reporter was transfected together with the Renilla plasmid into the cells. Luciferase activity was measured after 24 h.

### Co-Immunoprecipitation Assay

Immunoprecipitation was performed as described in previous study. The MCF-7 total cell lysls were pre-cleared with rabbit IgG for 2 h and subsequently immunoprecipitated with ERα antibody (D8H8, #8644) over night, while rabbit IgG (Santa Cruz) was used as the negative control. The bounded protein was analyzed by Anti-HOIL-1 antibody (Ab108479). For the overexpression experiment, HEK293 cells were transfected with 5 ug FLAG-HOIL-1 (Full length or deletion domains) and ERα plasmid (Full length or deletion domains) in 10 cm dish. Cell lysates were pre-cleared with IgG and subsequently incubate with Flag (Ab49763) antibody, while rabbit IgG was used as the negative control. The bound proteins were analyzed by western blotting.

### Poly-Ubiquitination Detection Assay

To directly detect the enriched overall ubiquitinated or K63-ubiqutinated ERα from the cell extracts, HEK293 cells were transfected with 4 ug Ub or 4 ug K63 Ubi plasmid, 2 ug ERα together with 0.5 ug Flag-HOIL-1 or Flag-vector. After 48 h, total protein was extracted and pre-cleared with 20 ul protein A (santa cruz, SC-2001) for 2 h. The supernatant was collected and immunoprecipitated by ERα antibody. Western blot with HA antibody was performed to detect K48, K63 poly-ubiquitinated or mono-ubiquitinated ERα.

### Immunofluorescence Assay

MCF-7 cells were fixed with 4% paraformaldehyde in PBS for 10 min, permeabilized with 0.2% Triton X-100 for 5 min, and blocked by 5% BSA in PBS for 1 h. A rabbit anti-HOIL-1 polyclonal antibody (Ab108479) and mouse anti-ERα monoclonal antibodies (SC-56833) were used, followed by Alexa Flour 647 (Invitrogen) anti-rabbit antibody and FITC-conjugated anti-mouse antibodies (Jackson ImmunoResearch, West Grove, PA). As negative controls, the samples were incubated with the secondary antibodies without primary antibodies. Images were acquired under conditions fulfilling the Nyquist criterion using Nikon A+ laser scanning confocal system with a 60× oil NA1.4 objective and pinhole size of 1.0 Airy Unit. The acquired pictures were further processed and assembled using ImageJ.

### Statistics

Student’s t-test, Pearson correlation coefficient, and Cox regression analysis were used for comparisons. A P-value of <0.05 was considered to be significant.

## Results

### HOIL-1 Is Elevated in Breast Cancer and Relates to Short Endocrine Treatment Outcome in Human Breast Cancer Tumors

**Figure 1:**
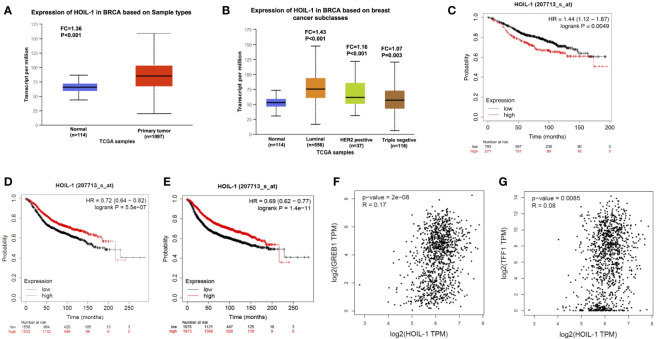
HOIL-1 is elevated in breast cancer and relates to short endocrine treatment outcome in human breast cancer tumors. **(A)** HOIL-1 mRNA level is elevated in breast cancer samples compared with breast tissues from TCGA database (https://tcga-data.nci.nih.gov). **(B)** HOIL-1 mRNA level is elevated in all subtypes of breast cancer compared with normal breast tissues from TCGA database (https://tcga-data.nci.nih.gov). **(C)** HOIL-1 mRNA level correlates with good prognosis in breast cancer patients. These clinical data are acquired from KMPLOT database (http://kmplot.com/analysis/). **(D)** HOIL-1 mRNA level correlates with good prognosis in ER positive breast cancer patients. These clinical data are acquired from KMPLOT database (http://kmplot.com/analysis/). **(E)** HOIL-1 mRNA level correlates with poor prognosis in endocrine-treated breast cancer patients. These clinical data are acquired from KMPLOT database (http://kmplot.com/analysis/). **(F)** HOIL-1 mRNA level correlates with GREB1 expression in human breast cancer samples. These clinical data are acquired from TCGA database (https://tcga-data.nci.nih.gov). **(G)** HOIL-1 mRNA level correlates with TFF1 expression in human breast cancer samples. These clinical data are acquired from TCGA database (https://tcga-data.nci.nih.gov).

We firstly analyzed the HOIL-1 expression level from public available database. The TCGA database (https://tcga-data.nci.nih.gov) showed that HOIL-1 was increased in breast cancer compared with breast tissues (Fold change = 1.35; P <0.001) (**Figure 1A**). In breast cancer subtype analysis, the data showed that HOIL-1 is all breast subtypes, including luminal type, HER2 positive type and triple negative type (**Figure 1B**). When we analyzed the HOIL-1 effect on breast cancer patient survival from KMPLOT database (https://kmplot.com), we observed that HOIL-1 related to longer progression survival in all patents and luminal type patients (**Figures 1C, D**). HIOL-1 expression also correlated with good prognosis in triple negative breast cancers (**Figure S1A**). However, HOIL specially correlated with poor survival in endocrine-treated patients (**Figure 1E**). Besides, the gene expression analysis from the TCGA database showed that HOIL-1 was positively correlated with ERα target gene expression including GREB1 and TFF1 in breast tumors (P <0.01, R = 0.17; P = 0.008, R = 0.08 respectively) (**Figures 1F, G**). These clinical data showed the consistent trend with previous reports that HOIL-1 might promote ERα signaling and endocrine resistance (23, 24).

### HOIL-1 Depletion Inhibits ERα Protein and ERα Signaling in Breast Cancer Cells

**Figure 2:**
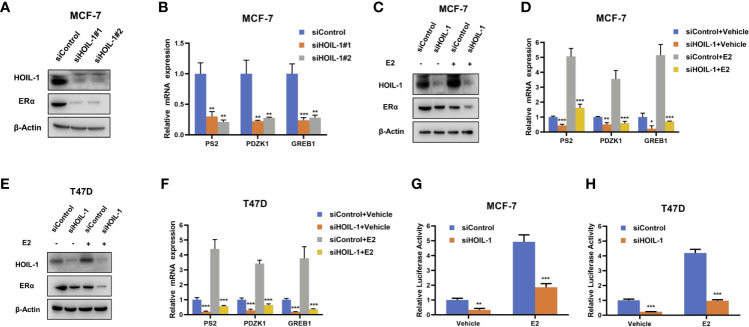
HOIL-1 depletion inhibits ERα protein and ERα signaling in breast cancer cells. **(A)** HOIL-1 depletion effect on ERα protein level by two different siRNA oligos. MCF-7 cells were transfected with two independent HOIL-1 siRNAs or siControl. HOIL-1 and ERα protein levels were determined by Western blot analysis. Tubulin was used as internal control. **(B)** HOIL-1 depletion decreases ERα target genes using two different siRNA oligos. MCF-7 cells were transfected with siHOIL-1 or siControl. After 48 h, total RNA was prepared and the expression of the endogenous ERα target genes, PS2, GREB1, and PDZK1 were determined by qPCR. Shown are the results from three experiments. *P <0.05; **P <0.01; ***P <0.001 for target gene expression comparison. **(C)** HOIL-1 depletion effect on ERα protein level. MCF-7 cells were transfected with siHOIL-1 or siControl. After 48 h, cells were treated with either ethanol or 10 nM estradiol for 6 h. HOIL-1 and ERα protein levels were determined by Western blot analysis. Tubulin was used as internal control. **(D)** HOIL-1 depletion effect on ERα protein level. T47D cells were transfected with siHOIL-1 or siControl. After 48 h, cells were treated with either ethanol or 10 nM estradiol for 6 h. HOIL-1 and ERα protein levels were determined by Western blot analysis. Tubulin was used as internal control. **(E)** HOIL-1 depletion decreases ERα target genes. MCF-7 cells were transfected with siHOIL-1 or siControl. After 48 h, cells were treated with either ethanol or 10 nM estradiol for 6 h. Total RNA was prepared and the expression of the endogenous ERα target genes, PS2, GREB1, and PDZK1 were determined by qPCR. Shown are the results from three experiments. *P <0.05; **P <0.01; ***P <0.001 for target gene expression comparison. **(F)** HOIL-1 depletion decreases ERα target genes. T47D cells were transfected with siHOIL-1 or siControl. After 48 h, cells were treated with either ethanol or 10 nM estradiol for 6 h. Total RNA was prepared and the expression of the endogenous ERα taarget genes, PS2, GREB1, and PDZK1 were determined by qPCR. Shown are the results from three experiments. *P <0.05; **P <0.01; ***P <0.001 for target gene expression comparison. **(G)** HOIL-1 depletion affects ERE-luciferase activity in MCF-7 cells. MCF-7 cells were transfected with siHOIL-1 or siControl together with ERE luciferase reporter plasmid. Cells were treated with 10 nM estradiol or vehicle. Luciferase activity was measured 48 h after transfection. Shown are the results from three experiments. *P <0.05; **P <0.01; ***P <0.001 for luciferase activity comparison. **(H)** HOIL-1 depletion affects ERE-luciferase activity in T47D cells. T47D cells were transfected with siHOIL-1 or siControl together with ERE luciferase reporter plasmid. Cells were treated with 10 nM estradiol or vehicle. Luciferase activity was measured 48 h after transfection. Shown are the results from three experiments. *P <0.05; **P <0.01; ***P <0.001 for luciferase activity comparison.

In order to uncover the role of HOIL-1 in ERα signaling in breast cancer cells, we depleted HOIP in MCF-7 cells. HOIL-1 depletion *via* two independent siRNA showed that HOIL-1 knocking-down decreases ERα protein level and ERα target gene expression, including PS2, GREB1 and PDZK1 (**Figures 2A, B**). Besides, HOIL-1 depletion could decrease ERα protein level in both vehicle and E2-treated condition in MCF-7 and T47D cells (**Figures 2C, D**). Consistent with this, HOIL-1 depletion also decreased ERα target gene expression, such as PS2, GREB1 and PDZK1 in both MCF7 and T47D cells (**Figures 2E, F**). In order to determine if HOIL-1 knocking down could affect ERα transcriptional activity, we measured estrogen response element (ERE) luciferase activity in both MCF-7 and T47D cells. It showed that HOIL-1 depletion decreases ERE luciferase activity in both MCF7 and T47D cells (**Figures 2G, H**). All these data indicate HOIL-1 is required for ERα signaling in breast cancer cells. In addition, we also found HOIL-1 depletion facilitated P53 protein level and its target gene expression (**Figures S1B, C**). We further investigated the function of HOIL-1 in ER negative breast cancer cells. In MDAMB231 cells, HOIL-1 depletion inhibited cell invasion (**Figures S2A–C**). Besides, HOIL-1 depletion also inhibited cell proliferation and migration in MDAMB231 cells (**Figures S2D, E**).

### HOIL-1 Is Mainly Localized in the Cytoplasm and Modulates ERα Stability

**Figure 3:**
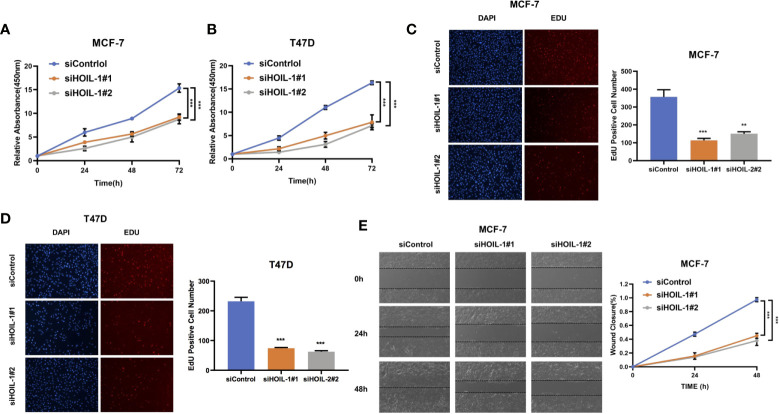
HOIL-1 is required for breast cancer progression in ER positive breast cancer cells. **(A)** HOIL-1 depletion inhibits the cell proliferation in breast cancer cells. MCF-7 cells were transfected with 50 nM HOIL-1 siRNA (mix of #1 and #2) or 50n M control siRNA. After 24 h, the WST assay was used to determine the cellar metabolic activity at indicated time points after transfection. Experiments were done in triplicates. *P <0.05; **P <0.01; ***P <0.001 for cell growth comparison. **(B)** HOIL-1 depletion inhibits the cell proliferation in breast cancer cells. T47D cells were transfected with 50 nM HOIL-1 siRNA (mix of #1 and #2) or 50 nM control siRNA. After 24 h, the WST assay was used to determine the cellar metabolic activity at indicated time points after transfection. Experiments were done in triplicates. *P <0.05; **P <0.01; ***P <0.001 for cell growth comparison. **(C)** HOIL-1 depletion decreased the EdU positive cells in MCF-7 cell. MCF-7 cells were transfected with 50 nM HOIL-1 siRNA (mix of #1 and #2) or 50 nM control siRNA. After 24 h, the EdU reagents were added into the cell culture medium for 2 h. After that, the cells were fixed and the EdU positive cells were counted. Experiments were done in triplicates. *P <0.05; **P <0.01; ***P <0.001 for comparison. **(D)** HOIL-1 depletion decreased the EdU positive cells in T47D cell. T47D cells were transfected with 50 nM HOIL-1 siRNA (mix of #1 and #2) or 50 nM control siRNA. After 24 h, the EdU reagents were added into the cell culture medium for 2 h. After that, the cells were fixed and the EdU positive cells were counted. Experiments were done in triplicates. *P <0.05; **P <0.01; ***P <0.001 for comparison. **(E)** Wound-healing assay of MCF-7 cells were transfected with siControl or siHOIL-1. Quantification of wound closure at the indicated time points. Data are presented as ± SD. **P <0.01, ***P < 0.001. * means the P value is less than 0.05 but more than 0.01.

In order to investigate the role of HOIL-1 in breast cancer cells, we depleted HOIL-1 in both MCF-7 and T47D cells. WST assay showed that HOIL-1 depletion significantly decreased breast cancer cell proliferation in MCF-7 and T47D cells In MTT assay (**Figures 3A, B**). Besides, the EdU (5-ethynyl-2-doxyuridine) incorporation assay showed that HOIL-1 depletion significantly decreased the EdU positive cells in MCF-7 and T47D cells (**Figures 3C, D**). In the wound-healing assay, we found that HOIL-1 was required for breast cancer cell migration in MCF-7 cells (**Figure 3E**).

**Figure 4:**
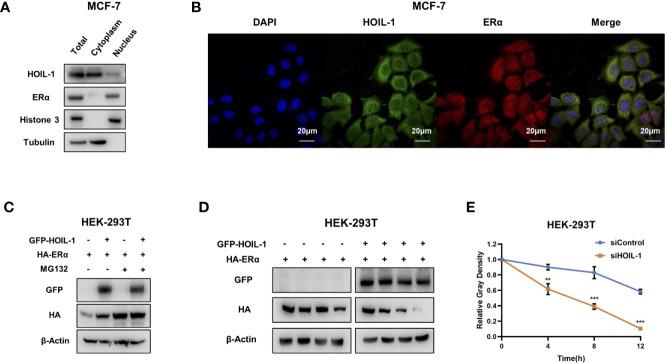
HOIL-1 is mainly localized in the cytoplasm and modulates ERα stability. **(A)** HOIL-1 protein mainly locates in the cytosol. The subcellular protein fractionation kit (Thermo scientific, 78840) was used for cytoplasm and nuclear separation. Tubulin and Histone-3 were used for cytoplasm and nuclear control. **(B)** Intracellular localization analysis of HOIL-1 and ERα by immunofluorescence assay. MCF7 cells were cultured in normal medium before fixation. Intracellular localization of HOIL-1 (green) and ERα (red) were shown. Nuclei (blue) were stained with 4’,6-diamidino-2-phenylindole (DAPI). **(C)** In the presence of the proteasome inhibitor MG132, the stabilization effect of HOIL-1 on ERα did not further increase ERα protein levels. HEK293 cells were transfected with 2 µg ERα plasmid and 0.5 µg Flag-tag or Flag-HOIL-1 plasmids. After 24 h, cells were treated with 10 uM MG132/vehicle for 6 h. Cell lysates were prepared for Western blot analysis. The results are representative for three independent experiments. **(D, E)** HOIL-1 increases ERα half-life in HEK293 cells. HEK293 cells were transfected with HA-ERα plasmid and Flag or Flag-HOIL-1 plasmids. After 24 h, cells were treated with 100 µM cycloheximide/vehicle for indicated times. Cell lysates were prepared for Western blot analysis. The results are representative for three independent experiments. The ERα relative density was measured by Image J software. ** means the P value is less than 0.01, but more than 0.001. *** means P value is less than 0.001.

Based on the significant impact in breast cancer cell phenotype by HOIL-1, we further investigated the potential mechanism. Nuclear and cytoplasm separation showed that HOIL-1 is mainly localized in the cytoplasm, while ERα is mainly localized in the nuclear (**Figure 4A**). Immuno-staining showed the same trend that ERα is located mainly in the nuclear, while HOIL-1 is mainly in the cytoplasm (**Figure 4B**). Then we investigated the potential role of HOIL-1 on ERα stability. Since ERα could regulate its own expression in MCF-7 cells, which make it difficult to identify the direct effect of HOIL-1 on ERα protein or mRNA (25). We utilize HEK293 cells to measure the protein stability of ERα *via* co-transfection with HOIL-1. In the protein stability assay, HOIL-1 could stabilize ERα. However, with the presence of a proteasome inhibitor MG132, the stabilization effect on ERα protein level could not further been increased (**Figure 4C**). With the inhibition of protein synthesis cycloheximde, HOIL-1 could significantly increases ERα stability in HEK293 cells (**Figures 4D, E**). All these data indicate that HOIL-1 could prolong ERα stability.

### HOIL-1 Associates With ERα AF1 Domain Through Its RING Domain and Stabilizes ERα Possibly by Promoting Mono-Ubiquitination

**Figure 5:**
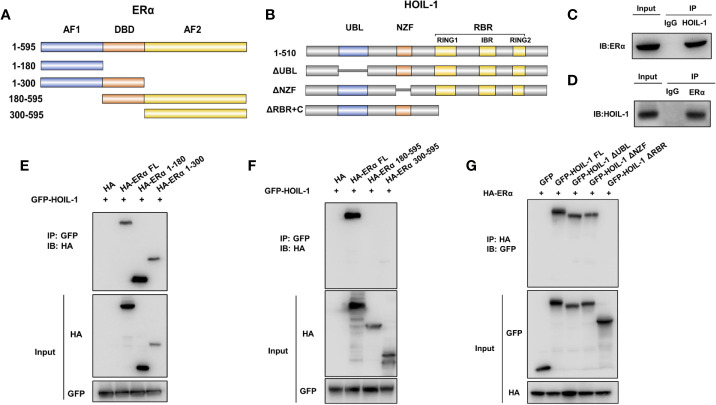
HOIL-1 associates with ERα AF1 domain through its RING domain and stabilizes ERα possibly by inhibition K48-linked ubiquitination. **(A)** ERα domain structure and deletion mutants used in the study (Full length, ΔAF1, ΔAF1 + ΔDBD, ΔAF2, ΔAF2 + ΔDBD). **(B)** HOIL-1 full length and deletion mutants are used in the study (Full length, ΔRBR, ΔNZF, ΔUBL domains). **(C, D)** Co-IP assay reveals association between endogenous HOIL-1 and ERα in MCF7 cells. MCF-7 cells were harvested with RIPA lysis buffer. CO-IP was performed using antibody as indicated. **(E, F)** HOIL-1 interacts with ERα through its AF1 domain. HEK293 cells were transfected with 2 µg Flag-HOIL-1 together with HA-ERα full length or mutants (ΔAF1, ΔAF1 + ΔDBD, ΔAF2 and ΔAF2 + ΔDBD). After 24 h, cells were harvested with NP-40 lysis buffer. CO-IP was performed using Flag antibody. The possible interacted ERα domains were detected by HA antibody. **(G)** RBR domain is required for HOIL-1 to interaction with ERα. HEK293 cells were transfected with 2 µg HA-ERα together with Flag-HOIL-1 full length or mutants (ΔRBR, ΔNZF, ΔUBL domain). After 24 h, cells were harvested with NP-40 lysis buffer. CO-IP was performed using HA antibody. The possible interacted HOIL-1 domains were detected by Flag antibody.

**Figure 6:**
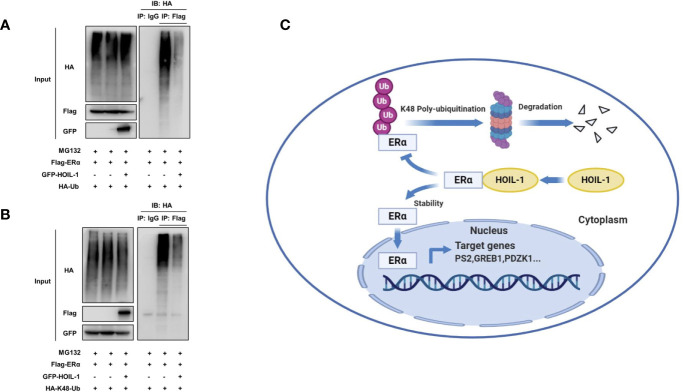
HOIL-1 enhances ERα stability through inhibiting ERα K48-linked poly-ubiquitination. **(A)** HOIL-1 prohibits ERα poly-ubiquitination. HEK293 cells were transfected with 2 µg ERα plasmid and 0.5 µg Flag or Flag-HOIL-1 plasmids. After 24 h, cells were treated with 10 uM MG132 for 6 h. Cells were directly harvested and Western blot analysis using ERα antibody was used to detect ubiquitinated ERα forms. The predicted molecular weight of polyubiquitinated ERα is indicated. **(B)** HOIL-1 decreases K48-linked poly-ubiquitination of ERα. HEK293 cells were transfected with 2 µg ERα plasmid, 0.5 µg HA-K48 Ubi plasmid and 0.5 µg Flag-HOIL-1 plasmids. The cell extracts were immunoprecipitated with HA antibody. The K48 specific poly-ubiquitinated ERα was detected *via* western blotting analysis. **(C)** The hypothetical model for HOIL-1 regulating ERα signaling in breast cancer cells: HOIL-1 associates with ERα *via* its RBR domain and stabilizes ERα protein probably *via* inhibiting ERα K48-linked poly-ubiquitination.

ERα is composed of three functional domains: AF1 domain, DNA binding domain and AF2 domain (**Figure 5A**). HOIL-1 is composed of three function domains, including UBL domain, NZF domain and RBR domain (**Figure 5B**). Endogenous immuno-precipitation shows that HOIL-1 could associate with ERα in MCF-7 cells (**Figures 5C, D**). Then the full length of ERα or ERα deletion constructs is transfected together with HOIL-1 in HEK293 cells. Co-IP indicates that HOIL-1 associates with ERα AF1 domain (**Figures 5E, F**). Besides, the full length of HOIL-1 or HOIL-1 deletion constructs is transfected together with ERα full length. Co-IP shows that ERα associates with HOIL-1 RBR domain (**Figure 5G**). Further experiments are carried out to measure ERα ubiquitination. Ubiquitin-based immuno-precipitation assay show that HOIL-1 could inhibit ERα overall ubiquitination (**Figure 6A**). K48-linked ubiquitin assay shows that HOIL-1 could inhibit ERα K48-linked ubiquitination (**Figure 6B**).

## Discussion

In this study, we identified the RING family E3 ubiquitin ligase HOIL-1, which was highly expressed in human breast cancer samples, facilitated ERα signaling and breast cancer progression *via* post-translational modification. HOIL-1 associated with ERα and inhibits ERα poly-ubiquitination and degradation (**Figure 6C**).

ERα was firstly cloned from MCF-7 cell in 1985 (26). About 70% of breast cancers are ERα positive, while the risk of breast caner is also correlated with the ERα expression level in breast tissue (27). Higher levels of ERα expression in breast cancer cell can lead to increased estrogen-independent activity of ERα (28). ER-positive cancer depends on ERα signaling for cell growth, which makes ERα a suitable target for breast cancer therapy. For ERα positive breast cancer patients, selective estrogen receptor modulators, such as tamoxifen, are standard endocrine treatment. However, endocrine resistance is one important issue in breast cancer therapy. Interesting, still most of the endocrine resistant breast cancer are ERα positive, which might indicate that ERα might play important role in mediating tamoxifen resistance. Modulating ERα protein stability could be one plausible strategy to overcome endocrine resistance.

There are about 500–1,000 different E3 ubiquitin ligases. Among these families, RING family is the largest. RING-In-Between-RING (RBR) E3 ligase is a subfamily of the RING family (29). One of the important functions of RBRs is the modulation of NFKB signaling and nuclear receptors (30–32). Recent studies reveal that several RBRs are necessary for ERα signaling activation and breast cancer proliferation. For example, RNF8 could associate with ERα and functions as a co-activator for ERα target genes. Besides, RNF8 could also mono-ubiquitinate ERα and promote ERα protein stability (17). Our previous work focused on several E3 ubiquitin ligases, which were able to enhance ERα signaling activity either *via* genomic regulation or post-translational modifications, including RNF31, RNF168 and SMURF1 (9, 20). Here, we identifies HOIL-1, which is one interaction protein with RNF31 could modulate ERα stability *via* inhibiting ERα poly-ubiquitination.

HOIL-1 was firstly identified in a yeast two-hybrid screen as a PKC interaction protein (33). The C-terminal part of the protein contains the RBR domain, while the N-terminal contains UBL domain and RZF domain (34). The RBR domain was regarded as functional domain for ubiquitin ligation, while the UBL domain could interact with 26 proteasome (35). Previous studies showed that HOIL1- could be an important marker for poor tamoxifen response (24). Nina et al. showed HOIL-1 could promoter ERα gene expression and also co-located with ERα at ERα target gene promoter regions (23). However, our study confirms that HOIL-1 is a positive modulator for ERα signaling, but through different mechanisms. Our immuno-staining indicates that HOIL-1 is mainly localized in the cytosol, not in the nuclear in MCF-7 cells. Since ERα is mostly degraded in the cytosol, HOIL-1 could exert its dual function in ERα signaling. When HOIL-1 in the nuclear, it co-activates ERα gene expression, while HOIL-1 is in the cytosol, it associates with ERα and enhances ERα stability.

Our study identifies that the RBR family protein HOIL-1 could modulate ERα signaling and breast cancer progression through a post-translational manner. Our study strengthens the critical role of HOIL-1 in ERα signaling and improves the understanding of HOIL-1 in both genomic regulation and post-translational regulation of ERα pathway. As such an important regulator of ERα signaling, HOIL-1 could be an important target for ERα positive breast cancer therapy.

## Data Availability Statement

The raw data supporting the conclusions of this article will be made available by the authors, without undue reservation.

## Author Contributions

JD performed most of the bench work. PK supervised the process of the study and performed the manuscript writing. JD participated in western blot and real-time PCR work. PK performed the prognosis data analysis. All authors contributed to the article and approved the submitted version.

## Funding

The project is supported by the Young Scientist grant of Nanchang University (PK).

## Conflict of Interest

The authors declare that the research was conducted in the absence of any commercial or financial relationships that could be construed as a potential conflict of interest.

## Supplementary Material

Supplementary Figure 1
**(A)** HOIL-1 mRNA level correlates with good prognosis in triple negative breast cancer patients. These clinical data are acquired from KMPLOT database (http://kmplot.com/analysis/). **(B)** HOIL-1 depletion effect on P53 protein level. MCF-7 cells were transfected with siHOIL-1 or siControl. After 48 h, HOIL-1 and P53 protein levels were determined by Western blot analysis. Actin was used as internal control. **(C)** HOIL-1 depletion increases P53 target genes. MCF-7 cells were transfected with siHOIL-1 or siControl. After 48 h, total RNA was prepared and the expression of the endogenous P53 target genes, P21, BTG2 and P53INP1 were determined by qPCR. Shown are the results from three experiments. *P<0.05; ** P<0.01; ***P<0.001 for target gene expression comparison.

Supplementary Figure 2
**(A)** HOIL-1 depletion effect on MDAMB231 cells by two different siRNA oligos. MDAMB231 cells were transfected with two independent HOIL-1 siRNAs or siControl. HOIL-1 protein levels were determined by Western blot analysis. Actin was used as internal control. **(B-C)** HOIL-1 depletion inhibited cancer cell invasion by trans-well assay in MDAMB231 cells. **(D)** HOIL-1 depletion inhibits the cell proliferation in breast cancer cells. MDAMB231 cells were transfected with 50nM HOIL-1 siRNA (mix of #1 and #2) or 50nM control siRNA. After 24 hours, the WST assay was used to determine the cellar metabolic activity at indicated time points after transfection. Experiments were done in triplicates. *P<0.05; ** P<0.01; ***P<0.001 for cell growth comparison. **(E-F)** Wound-healing assay of MDAMB231 cells were transfected with siControl or siHOIL-1. Quantification of wound closure at the indicated time points. Data are presented as ± SD. **, P<0.01, ***, P< 0.001.

## Abbreviations

HOIL-1, Homologous to the E6-AP Carboxyl Terminus 1; AF1, Transcriptional activation domain 1; DBD, DNA binding domain; AF2, Transcriptional activation domain 2; ERα, Estrogen receptor alpha; HER2, Human epidermal growth factor receptor 2; PR, Progesterone receptor; TBNC, Triple negative breast cancer; RBR, RING-between-RING domain; NZF, Npl4 zinc finger domain; EMT, Epithelial–mesenchymal transition; ATCC, American Type Culture Collection.
