# Supplementary figures and images for: Regulation of ERα Stability and Estrogen Signaling in Breast Cancer by HOIL-1
Source: Front Oncol. 2021 May 20;11:664689. doi: 10.3389/fonc.2021.664689 (PMC8173209; doi:10.3389/fonc.2021.664689)

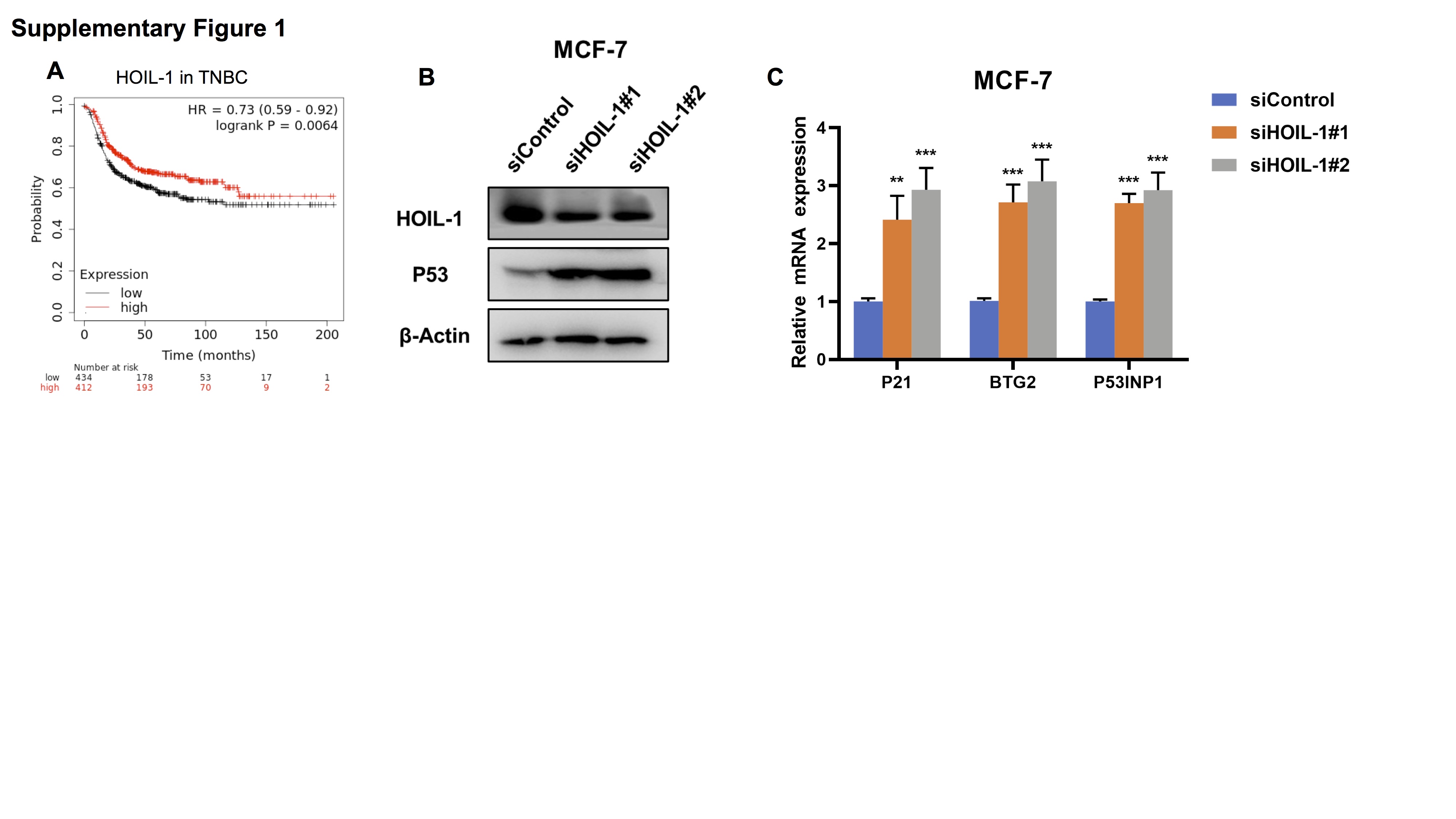

Supplement: Supplementary Figure 1 — (A) HOIL-1 mRNA level correlates with good prognosis in triple negative breast cancer patients. These clinical data are acquired from KMPLOT database (http://kmplot.com/analysis/). (B) HOIL-1 depletion effect on P53 protein level. MCF-7 cells were transfected with siHOIL-1 or siControl. After 48 h, HOIL-1 and P53 protein levels were determined by Western blot analysis. Actin was used as internal control. (C) HOIL-1 depletion increases P53 target genes. MCF-7 cells were transfected with siHOIL-1 or siControl. After 48 h, total RNA was prepared and the expression of the endogenous P53 target genes, P21, BTG2 and P53INP1 were determined by qPCR. Shown are the results from three experiments. *P<0.05; ** P<0.01; ***P<0.001 for target gene expression comparison. [file Image_1.jpeg]

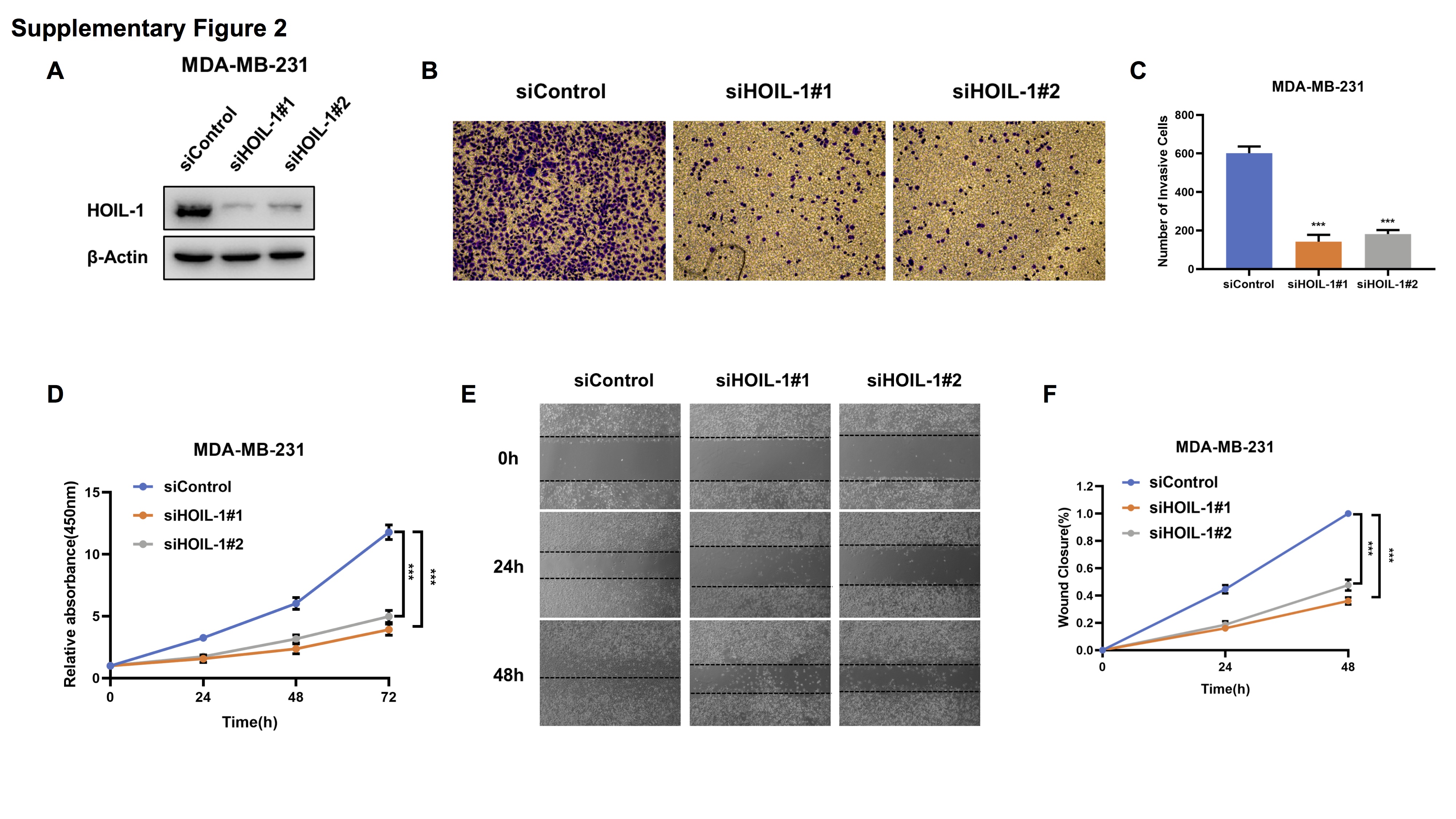

Supplement: Supplementary Figure 2 — (A) HOIL-1 depletion effect on MDAMB231 cells by two different siRNA oligos. MDAMB231 cells were transfected with two independent HOIL-1 siRNAs or siControl. HOIL-1 protein levels were determined by Western blot analysis. Actin was used as internal control. (B-C) HOIL-1 depletion inhibited cancer cell invasion by trans-well assay in MDAMB231 cells. (D) HOIL-1 depletion inhibits the cell proliferation in breast cancer cells. MDAMB231 cells were transfected with 50nM HOIL-1 siRNA (mix of #1 and #2) or 50nM control siRNA. After 24 hours, the WST assay was used to determine the cellar metabolic activity at indicated time points after transfection. Experiments were done in triplicates. *P<0.05; ** P<0.01; ***P<0.001 for cell growth comparison. (E-F) Wound-healing assay of MDAMB231 cells were transfected with siControl or siHOIL-1. Quantification of wound closure at the indicated time points. Data are presented as ± SD. **, P<0.01, ***, P< 0.001. [file Image_2.jpeg]
